# Incidences of obesity and extreme obesity among US adults: findings from the 2009 Behavioral Risk Factor Surveillance System

**DOI:** 10.1186/1478-7954-9-56

**Published:** 2011-10-17

**Authors:** Liping Pan, David S Freedman, Cathleen Gillespie, Sohyun Park, Bettylou Sherry

**Affiliations:** 1 Division of Nutrition, Physical Activity, and Obesity, National Center for Chronic Disease Prevention and Health Promotion, Centers for Disease Control and Prevention, Atlanta, GA, USA; 2 Division for Heart Disease and Stroke Prevention, National Center for Chronic Disease Prevention and Health Promotion, Centers for Disease Control and Prevention, Atlanta, GA, USA

**Keywords:** Body Mass Index, Obesity, Incidence, Risk Prediction

## Abstract

**Background:**

No recent national studies have provided incidence data for obesity, nor have they examined the association between incidence and selected risk factors. The purpose of this study is to examine the incidence of obesity (body mass index [BMI] ≥ 30.0 kg/m^2^) and extreme obesity (BMI ≥ 40.0 kg/m^2^) among US adults and to determine variations across socio-demographic characteristics and behavioral factors.

**Methods:**

We used a weighted sample of 401,587 US adults from the 2009 Behavioral Risk Factor Surveillance System. Incidence calculations were based on respondent's height and current and previous weights. Logistic regression was used to examine associations between incidence and selected socio-demographic characteristics and behavioral factors.

**Results:**

The overall crude incidences of obesity and extreme obesity in 2009 were 4% and 0.7% per year, respectively. In our multivariable analyses that controlled for baseline body mass index, the incidences of obesity and extreme obesity decreased significantly with increasing levels of education. Incidences were significantly higher among young adults, women, and adults who did not participate in any leisure-time physical activity. Incidence was lowest among non-Hispanic whites.

**Conclusions:**

The high incidence of obesity underscores the importance of implementing effective policy and environmental strategies in the general population. Given the significant variations in incidence within the subgroups, public health officials should prioritize younger adults, women, minorities, and adults with lower education as the targets for these efforts.

## Background

The prevalence of obesity among adults has more than doubled in the past three decades, and obesity continues to be a public health concern [1,2]. One of the objectives of Healthy People 2020 is to reduce the proportion of adults who are obese [3]. It is well established that obesity is related to reduced quality of life, increased risk for premature death, and increased risk for many chronic diseases, including coronary heart disease, hypertension, stroke, Type 2 diabetes, and certain types of cancer [4,5]. Obesity is also associated with increased health care costs. It has been estimated that obesity was associated with almost 10% of annual medical spending and that obesity-related medical costs reached $147 billion in 2008 [6].

Numerous studies have examined the prevalence of obesity and extreme obesity, with findings indicating that more than one-third of US adults were obese during 2007-2008 [2,7]. Prevalence data are very useful to identify high risk populations for interventions; however, prevalence focuses on people who have been obese or extremely obese for amounts of time, as well as those who have recently attained such status. Prevalence indicates the magnitude of the problem of obesity, whereas incidence conveys information about the rate of developing it. Incidence identifies those who become obese or extremely obese over a certain period of time. Incidence data are valuable because they enable elucidation of the characteristics of the incident cases during the study period, thus identifying those at high risk for becoming obese to target for prevention efforts. Reducing obesity prevalence requires interventions to help those who are already obese maintain or lose weight as well as interventions to prevent new cases from becoming obese. Several studies have examined the incidence of obesity between the 1950s and the early 2000s and identified demographic characteristics associated with the incidence [8-10]. However, no recent national studies have provided incidence data, nor have they examined the association between incidence and selected risk factors. Because various factors, such as age, sex, race, physical activity, and smoking, have all been associated with the prevalence of obesity [2,11,12], we assumed that these factors also may be related to the incidence of obesity. The purpose of our study is to examine the incidences of obesity and extreme obesity, socio-demographic characteristics and behavioral factors associated with these incidences, and to examine changes in BMI categories over a one-year period.

## Methods

### Data

We used data from the 2009 Behavioral Risk Factor Surveillance System (BRFSS). BRFSS is an ongoing, state-based, telephone interview survey conducted annually by the Centers for Disease Control and Prevention and state health departments. The survey is based on a multistage cluster design that uses random-digit dialing to select a sample that represents the civilian noninstitutionalized adult population in each of the 50 states, the District of Columbia, and three US territories. Post-stratification weights are used to adjust for nonresponse, noncoverage, and disproportionate selection of population, and to produce demographic distributions that correspond closely to the state population. Detailed descriptions of its sampling design and methods have been previously published [13,14].

We used a weighted sample of 432,607 US adults. We excluded subjects who reported a current or previous weight ≥ 500 pounds (n = 107) or a height ≥ 7 feet or < 3 feet (n = 32), subjects who were missing data for height (n = 5,599) or for current or previous weight (n = 22,103), subjects who had a current or previous body mass index (BMI, calculated from weight [kg]/height [m^2^]) less than the minimum BMI value calculated from measured heights and weights of the third and fourth National Health and Nutrition Examination Survey participants (BMI < 11.7 kg/m^2^, n = 55), women who reported that they were pregnant (n = 2,480), and women aged 18 to 44 years who did not report pregnancy status (n = 644), which yielded a final sample size of 401,587.

### Self-reported weight and height and incidence calculation

Obesity and extreme obesity were defined as having a BMI ≥ 30.0 kg/m^2 ^and ≥ 40.0 kg/m^2^, respectively [4]. Respondents were asked, "About how much do you weigh without shoes?", "How much did you weigh a year ago?", and "About how tall are you without shoes?" Current BMI was calculated from self-reported current weight and height. BMI in previous year was calculated from self-reported weight in previous year and current self-reported height. Weight changes were calculated by subtracting respondents' current weight from their previous weight.

We calculated the incidences of obesity and extreme obesity in various groups. The numerator was defined as adults who became obese or extremely obese between 2008 and 2009. The populations in the denominators were defined as those who were at risk of becoming obese (BMI 11.7-29.9 kg/m^2^) or extremely obese (BMI 11.7-39.9 kg/m^2^) in 2008. Incidences of obesity and extreme obesity were then calculated by dividing the numbers of adults who developed obesity or extreme obesity during the one-year period by the numbers of adults who were at risk. Among those included in the final sample, 284,122 respondents were at risk of becoming obese, and 382,713 respondents were at risk of becoming extremely obese. To fully understand weight changes, we also examined weight loss between 2008 and 2009.

### Socio-demographic characteristics and behavioral factors

For socio-demographic characteristics, we used four age groups (18-29, 30-49, 50-69, and ≥ 70 years), three racial/ethnic groups (non-Hispanic white, non-Hispanic black, and Hispanic), four levels of education (less than high school, high school, some college, and college graduate), and five regions (US territories composite and four US census regions: Northeast, Midwest, South, and West). For the 2008 baseline BMI, three categories (11.7-24.9 kg/m^2^, 25.0-27.4 kg/m^2^, and 27.5-29.9 kg/m^2^) were used to estimate adjusted incidence of obesity, and five categories (11.7-29.9 kg/m^2^, 30-32.4 kg/m^2^, 32.5-34.9 kg/m^2^, 35.0-37.4 kg/m^2^, 37.5-39.9 kg/m^2^) were used for adjusted incidence of extreme obesity to ensure adequate sample size.

For behavioral factors, we examined four areas: physical activity, fruit and vegetable consumption, smoking, and alcohol drinking. We used the leisure-time physical activity question, "During the past month, other than your regular job, did you participate in any physical activities or exercise such as running, calisthenics, golf, gardening, or walking for exercise?", as a proxy for physical activity. We created a dichotomous variable to measure fruit and vegetable consumption: < 5 times/day versus ≥ 5 times/day. Fruit and vegetable consumption was based on a six-item frequency screener concerning fruit juices, fruit, green salad, nonfried potatoes, carrots, and other vegetables. We measured smoking status by three groups: nonsmoker, former smoker, and current smoker. We also included three alcohol drinking categories: no drinking, any drinking (adults who have had at least one drink of any alcoholic beverage during the past month but are not heavy drinkers), and heavy drinking (adult men having > 2 drinks/day and adult women having > 1 drink/day).

For our analyses, we used 2009 data on current weight status, socio-demographic and behavioral variables, as well as weight change based on recall of weight a year ago. To examine the association of incidence with behavioral factors, we made the assumption that respondents' health behaviors did not change between 2008 and 2009, and their current behaviors were used as a proxy to predict incidence.

### Statistical analyses

All statistical analyses were carried out using SAS-Callable SUDAAN (Version 9.2; SAS Institute, Cary, North Carolina). The variances for the estimates were adjusted to the BRFSS complex sampling design, including stratification, primary sampling unit, and clustering.

We used descriptive statistics to examine the characteristics of the study sample and mean weight changes. We used t-tests to compare the differences in mean weight changes between groups and polynomial contrasts to test for linear trends in mean weight changes across level of education and baseline BMI. We conducted logistic regression to estimate the adjusted incidences of obesity and extreme obesity and to identify factors associated with incidences. Because of missing data on covariates, the models included 253,183 respondents who were at risk for obesity and 342,284 respondents who were at risk for extreme obesity. The covariates included in the models were age, sex, race/ethnicity, level of education, region, physical activity, fruit and vegetable consumption, smoking, alcohol drinking, and baseline BMI. We calculated predictive margins (adjusted incidence) for various groups, adjusting for all other variables in the model. Predictive margin is a type of direct standardization in which the predicted values from the logistic regression model are averaged over the covariate distribution of the population, allowing control for differences in covariate distribution between groups [15]. We calculated risk ratios by comparing the predictive margins for respondents across groups with certain socio-demographic and behavioral characteristics. In addition, we used pairwise contrast to examine the differences in adjusted incidence across groups and defined a linear contrast to test the presence of a linear trend across baseline BMI in each of the logistic regression models. To control for the inflated type 1 error rate due to our large sample size, we used p < 0.01 to define statistical significance.

## Results

### Description of respondents

The characteristics of our analytic sample are described in Table 1. Although our analytic sample included about 40% men and 60% women, it was weighted to represent the age, sex, and race distribution of the state's adult population. Approximately 69% of our weighted sample were non-Hispanic whites, 42% were 50 years or older, 35% had at least a college education, and 36% resided in the South.

**Table 1:** Descriptive statistics by socio-demographic characteristics, Behavioral Risk Factor Surveillance System: United States, 2009

Characteristic/behavioral factor	Men (N = 158,770)		Women (N = 242,817)	
	
	n	% ^a^	n	% ^a^
Age group (years)				
18-29	10,884	20.3	14,175	17.4
30-49	45,485	39.6	67,503	37.3
50-69	69,901	29.6	101,696	30.6
≥ 70	31,842	10.2	58,064	14.3
Race/ethnicity				
White, non-Hispanic	127,416	68.2	190,829	69.8
Black, non-Hispanic	10,064	9.1	20,847	10.4
Hispanic	10,019	14.4	16,343	13.3
Educational level				
< High school	14,261	10.5	21,997	9.6
High school	45,951	28.3	73,647	27.8
Some college	39,137	25.1	68,924	28.4
College graduate	59,176	35.9	77,931	34.1
Census region				
Northeast	28,747	17.7	43,762	18.0
Midwest	38,326	21.8	57,265	22.0
South	45,803	36.0	77,513	36.2
West	43,258	23.2	59,690	22.4
Territory	2636	1.2	4587	1.3

^a ^Weighted percentage; the percentages in each category may not add up to 100% because of missing data

### Mean weight changes

Among all the adults included in this study, the mean weight change between 2008 and 2009 was about a one pound weight loss (95% confidence interval [CI] = -1.2, -0.9 lb). Among those who were at risk of becoming extremely obese, the mean weight loss was 0.2 lb (-0.3, -0.1). However, the most weight loss occurred among those who were previously obese. Among adults who were at risk of becoming obese, there was a mean weight gain of 1.5 lb (1.4, 1.6). Among those who were at risk of becoming obese or extremely obese, the respondents with higher education levels and baseline BMI had significantly lower mean weight gain (p < 0.01), whereas young adults, women, non-Hispanic blacks, and Hispanics had significantly higher mean weight gain (p < 0.01) during the previous year (Table 2).

**Table 2:** Mean weight changes among adults aged ≥18 years who were at risk for obesity ^a ^and extreme obesity ^b ^between 2008 and 2009, by socio-demographic characteristic, Behavioral Risk Factor Surveillance System: United States, 2009

	At risk for obesity		At risk for extreme obesity	
	(BMI 11.7-29.9 kg/m^2^)		(BMI 11.7-39.9 kg/m^2^)	
	(N = 284,122)		(N = 382,713)	
	
Characteristic	Mean change (lb)^c^	(95% CI)	Mean change (lb)^c^	(95% CI)
Overall	1.5	(1.4, 1.6)	-0.2	(-0.3, -0.1)
Age group (years)				
18-29	3.9	(3.5, 4.2)	2.3	(1.9, 2.6)
30-49	1.7	(1.6, 1.9)	0.1	(-0.1, 0.2)
50-69	0.6	(0.5, 0.7)	-1.2	(-1.3, -1.1)
≥ 70	-1.0	(-1.2, -0.9)	-2.3	(-2.4, -2.1)
Sex				
Women	1.6	(1.5, 1.8)	0.2	(0.1, 0.3)
Men	1.3	(1.1, 1.5)	-0.5	(-0.7, -0.4)
Race/ethnicity				
White, non-Hispanic	0.9	(0.8, 1.0)	-0.6	(-0.7, -0.5)
Black, non-Hispanic	3.7	(3.1, 4.2)	1.2	(0.7, 1.6)
Hispanic	3.1	(2.7, 3.5)	1.0	(0.6, 1.4)
Educational level				
< High school	3.6	(3.2, 4.0)	1.4	(1.0, 1.8)
High school	2.2	(2.0, 2.4)	0.3	(0.1, 0.5)
Some college	1.5	(1.4, 1.7)	-0.3	(-0.5, -0.2)
College graduate	0.4	(0.3, 0.5)	-0.8	(-1.0, -0.7)
Census region				
Northeast	1.4	(1.2, 1.7)	-0.2	(-0.4, 0.1)
Midwest	1.4	(1.2, 1.5)	-0.3	(-0.5, -0.1)
South	1.7	(1.5, 1.9)	-0.1	(-0.2, 0.1)
West	1.3	(1.1, 1.5)	-0.2	(-0.4, 0.0)
Territory	1.9	(1.3, 2.4)	0.3	(-0.3, 0.9)
Baseline BMI (kg/m^2^)				
11.7-24.9	3.0	(2.8, 3.1)	- ^d^	- ^d^
25.0-27.4	0.6	(0.5, 0.8)	-	-
27.5-29.9	-1.2	(-1.4, -0.9)	-	-
11.7-29.9	-^e^	- ^e^	1.5	(1.4, 1.6)
30.0-32.4	-	-	-3.0	(-3.3, -2.7)
32.5-34.9	-	-	-5.5	(-6.0, -5.0)
35.0-37.4	-	-	-7.3	(-7.9, -6.6)
37.5-39.9	-	-	-10.1	(-11.2, -9.1)

^a ^At risk for obesity: BMI 11.7-29.9 kg/m^2^

^b ^At risk for extreme obesity: BMI 11.7-39.9 kg/m^2^

^c ^Weighted mean

^d ^Not applicable; we combined these three baseline BMI categories for adults who were at risk for extreme obesity to ensure adequate sample size

^e ^Not applicable; we used three baseline BMI categories (as indicted in the table) for adults who were at risk for obesity

To examine the distribution of weight change in this population, we reported both weight gain and weight loss. Some respondents experienced weight gain between 2008 and 2009 (Table 3). For example, 35.8% of the underweight (baseline BMI 11.7-18.4 kg/m^2^) adults achieved normal weight status, and 9.5% of the previously normal weight (baseline BMI 18.5-24.9 kg/m^2^) adults became overweight. During the same time, some other adults experienced weight loss and moved from higher to lower BMI categories (Table 3). For example, 16.7% of the previously obese (baseline BMI 30.0-39.9 kg/m^2^) adults became overweight, and 29.6% of the previously extremely obese (baseline BMI ≥ 40.0 kg/m^2^) adults became obese in 2009.

**Table 3:** Change of BMI status between 2008 and 2009 among adults aged ≥18 years, Behavioral Risk Factor Surveillance System

	2009 BMI (kg/m^2^) % ^a ^(standard error [SE])				
	
2008 BMI (kg/m^2^)	11.7-18.4	18.5-24.9	25.0-29.9	30.0-39.9	≥ 40.0
11.7-18.4	62.0 (1.37)	35.8 (1.37)	1.6 (0.32)	0.5 (0.13)	0.02 (0.02)
18.5-24.9	0.9 (0.05)	88.8 (0.19)	9.5 (0.18)	0.7 (0.06)	0.04(0.01)
25.0-29.9	0.1 (0.01)	9.0 (0.17)	83.4 (0.22)	7.5 (0.16)	0.1(0.03)
30.0-39.9	0.1 (0.02)	1.2 (0.07)	16.7 (0.26)	79.3 (0.29)	2.7 (0.13)
≥ 40.0	0.1 (0.08)	0.3 (0.04)	2.4 (0.22)	29.6 (0.71)	67.6 (0.72)

^a ^Weighted percentage, the row percentages sum to 100%

### Factors associated with incidence of obesity and extreme obesity

The overall crude incidences of obesity and extreme obesity in 2009 were 4.0% (3.9%, 4.2%) and 0.7% (0.6%, 0.8%) per year, respectively. In our multivariable analyses, the strongest factor associated with both the incidence of obesity and extreme obesity was baseline BMI. As the baseline BMI increased, the incidences of obesity and extreme obesity significantly increased (p < 0.01 for trend test across BMI) (Table 4).

**Table 4:** Adjusted incidence ^a ^and risk ratios of obesity ^b ^and extreme obesity ^c ^among adults aged ≥18 years, by selected socio-demographic characteristics and behavioral factors, Behavioral Risk Facto Surveillance System: United States, 2009

	Obesity (BMI ≥ 30.0 kg/m^2^)			Extreme obesity (BMI ≥ 40.0 kg/m^2^)		
Characteristic/behavioral factor	Adjusted incidence	Risk ratio		Adjusted incidence	Risk ratio	
	% (SE)	Ratio	(95% CI)	% (SE)	Ratio	(95% CI)
Age group (years)						
18-29	6.4 (0.37)	1.0 ^d^		1.2 (0.15)	1.0	
30-49	**4.8 (0.16) **^e^	0.8	(0.7, 0.9)	0.9 (0.06)	0.7	(0.6, 1.0)
50-69	**3.3 (0.11)**	0.5	(0.5, 0.6)	**0.5 (0.03)**	0.4	(0.3, 0.6)
≥ 70	**1.5 (0.10)**	0.2	(0.2, 0.3)	**0.2 (0.04)**	0.2	(0.1, 0.3)
Sex						
Women	5.4 (0.14)	1.0		0.9 (0.05)	1.0	
Men	**3.2 (0.12)**	0.6	(0.5, 0.6)	**0.6 (0.05)**	0.6	(0.5, 0.8)
Race/ethnicity						
White, non-Hispanic	3.4 (0.09)	1.0		0.6 (0.04)	1.0	
Black, non-Hispanic	**6.0 (0.41)**	1.8	(1.5, 2.0)	0.8 (0.08)	1.3	(1.1, 1.6)
Hispanic	**5.5 (0.37)**	1.6	(1.4, 1.9)	1.0 (0.15)	1.5	(1.1, 2.1)
Educational level						
< High school	5.1 (0.34)	1.0		0.9 (0.12)	1.0	
High school	4.8 (0.20)	0.9	(0.8, 1.1)	0.8 (0.07)	0.9	(0.6, 1.2)
Some college	4.2 (0.17)	0.8	(0.7, 1.0)	0.7 (0.06)	0.7	(0.5, 1.0)
College graduate	**2.9 (0.13)**	0.6	(0.5, 0.7)	**0.5 (0.05)**	0.5	(0.4, 0.7)
Census region						
Northeast	4.1 (0.22)	1.0		0.6 (0.07)	1.0	
Midwest	4.1 (0.16)	1.0	(0.9, 1.1)	0.7 (0.06)	1.1	(0.8, 1.4)
South	4.3 (0.17)	1.1	(0.9, 1.2)	**0.9 (0.07)**	1.4	(1.1, 1.8)
West	3.8 (0.21)	0.9	(0.8, 1.1)	0.6 (0.08)	0.9	(0.7, 1.3)
Territory	3.4 (0.42)	0.8	(0.6, 1.1)	0.3 (0.10)	0.5	(0.3, 1.0)
Leisure-time physical activity						
No	5.1 (0.19)	1.0		1.0 (0.07)	1.0	
Yes	**3.7 (0.11)**	0.7	(0.7, 0.8)	**0.6 (0.04)**	0.6	(0.5, 0.7)
≥ 5 times of fruits and vegetables per day						
No	4.1 (0.11)	1.0		0.7 (0.04)	1.0	
Yes	3.9 (0.18)	0.9	(0.8, 1.0)	0.6 (0.07)	0.9	(0.7, 1.1)
Smoking						
Nonsmoker	3.5 (0.11)	1.0		0.7 (0.05)	1.0	
Former smoker	**5.0 (0.20)**	1.4	(1.3, 1.6)	0.8 (0.07)	1.2	(1.0, 1.5)
Current smoker	**4.8 (0.23)**	1.4	(1.2, 1.6)	0.7 (0.08)	1.0	(0.8, 1.3)
Alcohol drinking						
No drinking	4.4 (0.14)	1.0		0.8 (0.05)	1.0	
Any drinking	**3.8 (0.14)**	0.9	(0.8, 1.0)	**0.6 (0.05)**	0.8	(0.6, 0.9)
Heavy drinking	3.8 (0.40)	0.9	(0.7, 1.1)	0.7 (0.20)	0.9	(0.5, 1.6)
Baseline BMI (kg/m^2^)						
11.7-24.9	0.7 (0.06)	1.0		-^f^	- ^f^	
25.0-27.4	**3.7 (0.19)**	5.5	(4.4, 6.8)	-	-	-
27.5-29.9	**14.4 (0.37)**	21.6	(17.8, 26.2)	-	-	-
11.7-29.9	-^g^	-^g^		0.1 (0.02)	1.0	
30.0-32.4	-	-	-	**0.5 (0.10)**	6.6	(3.6, 12.1)
32.5-34.9	-	-	-	**1.0 (0.14)**	13.9	(8.3, 23.5)
35.0-37.4	-	-	-	**3.4 (0.28)**	48.5	(30.2, 77.9)
37.5-39.9	-	-	-	**11.4 (0.85)**	161.6	(101.0, 258.7)

^a ^Predictive margins adjusted for baseline BMI and all other socio-demographic characteristics and behavioral factors in the model

^b ^Obesity: BMI ≥30.0 kg/m^2^

^c ^Extreme obesity: BMI ≥40.0 kg/m^2^

^d ^Reference group

^e ^p < 0.01 for pairwise contrast to test for the difference between the bolded estimate and the estimate for the reference group

^f ^Not applicable; we combined these three baseline BMI categories for adults who were at risk for extreme obesity to ensure adequate sample size

^g ^Not applicable; we used three baseline BMI categories (as indicted in the table) for adults who were at risk for obesity

However, even after controlling for baseline BMI, we found that the incidences of obesity and extreme obesity varied by socio-demographic characteristics (Table 4). These incidences were significantly higher among younger age groups. The incidences of obesity and extreme obesity among adults aged ≥ 70 years was more than 75% lower than the incidences among those aged 18-29 years (1.5% versus 6.4%, and 0.2% versus 1.2%, respectively, p < 0.01). Women had 50% or higher incidences of obesity and extreme obesity than did men (5.4% versus 3.2%, and 0.9% versus 0.6%, respectively, p < 0.01). Non-Hispanic whites and college graduates had lower incidences of obesity and extreme obesity. Adults in the South had a significantly higher incidence of extreme obesity than adults in the Northeast, West, and territories (p < 0.01), and adults living in the Midwest had a higher incidence of extreme obesity than adults living in the territories (p < 0.01) (Table 4).

After adjusting for other variables in the model, participating in any leisure-time physical activity was significantly associated with a 30% reduction in incidence of obesity and a 40% reduction in incidence of extreme obesity (Table 4). In comparison to nonsmokers, former and current smokers who were at risk for obesity had a 40% higher risk of becoming obese during the past year. Compared with no consumption, any consumption of alcohol during past month was associated with a 10% decreased risk for obesity and a 20% decreased risk for extreme obesity.

## Discussion

We found that the incidences of obesity and extreme obesity rose with increasing baseline BMI. The incidences of obesity and extreme obesity were higher among younger adults, women, non-Hispanic blacks and Hispanics, adults with lower levels of education, and adults who did not participate in any leisure-time physical activity. Adults who did not drink any alcoholic beverages had higher incidences than those who had at least one drink but were not heavy drinkers during the past month. Many, but not all, of these associations agree with the results of studies based on the prevalence of obesity. We also found that a considerable proportion of obese and extremely obese adults lost weight between 2008 and 2009 and moved to lower BMI categories. The mean weight loss increased as baseline BMI increased.

Baseline BMI was the most significant indicator for both obesity and extreme obesity incidence. This result was not surprising because adults with high baseline BMI would have had to gain less weight to become obese or extremely obese. This finding indicates that prevention of further weight gain should be the first step of obesity control, especially among "at-risk" adults with high baseline BMI, because obesity-related morbidities increase with increasing BMI [4,5].

The incidences of obesity and extreme obesity were highest among adults aged 18 to 29 years, indicating that young adults are more likely to develop a weight problem even though the prevalence of obesity is lowest among this group [7]. Obesity is associated with morbidity and with the leading causes of death in the United States [4,5]. The risk for obesity-related chronic diseases will be significantly increased among young adults, and their quality of life will be considerably diminished throughout the rest of their lives once they become obese. Therefore, obesity prevention efforts are likely to have the biggest impact on young adults in their 20s. Consistent with previous studies [16,17], we found that women were more likely to develop obesity than were men. These findings suggest that young adults, particularly young women, are important groups to focus on to prevent obesity.

Non-Hispanic blacks, Hispanics, and adults with lower levels of education had higher incidences of obesity and extreme obesity. Behavioral, cultural, and environmental factors may have contributed to the high incidences. According to one study, both non-Hispanic black women and Hispanic women are more satisfied with their body size than are non-Hispanic white women; those who are satisfied with their body size are less likely to try to lose weight [18]. Evidence also suggests that black, Hispanic, and lower-income neighborhoods have fewer chain supermarkets and produce stores and less access to physical activity facilities; this limited access may negatively impact diet and physical activity levels [19].

Using the same data source, the 2009 BRFSS, a previous study indicated that the South and Midwest had higher prevalences of obesity than the Northeast and West [7]. Our study shows that the South has a significantly higher incidence of extreme obesity than the Northeast, West, and the territories. The South may be a geographic region that warrants extra obesity prevention efforts.

Certain behavioral factors were associated with the incidences of obesity and extreme obesity even after controlling for baseline BMI and socio-demographic characteristics. Participating in any leisure-time physical activity was associated with decreased risks of developing obesity and extreme obesity. Physical activity plays a role in the maintenance of a healthy body weight, the loss of excess body weight, and the maintenance of successful weight loss because of its role in energy balance [11]. Increasing physical activity among US adults through informational, behavioral, and environmental evidence-based approaches is important for obesity prevention [20].

As indicated in our study, any alcohol drinking was related to a decreased risk for obesity and extreme obesity compared to no alcohol drinking. This finding was similar to the results from a prospective cohort study conducted by Wang and colleagues [21]. They concluded that normal weight middle-aged and older women who consumed a light to moderate amount of alcohol had a lower risk of becoming overweight and/or obese during 12.9 years of follow-up compared to nondrinkers. However, our finding should be interpreted with caution because our nondrinker group not only included those who never consume alcohol, but also former drinkers. The underlying mechanism for the association between obesity and alcohol consumption is complex and needs to be better understood. Studies found that some drinkers, especially female drinkers, tend to substitute alcohol for other foods without increasing total calorie intake, and lower intake of carbohydrates was related to higher levels of alcohol intake [21,22].

Similar to a cohort study conducted by Watari and colleagues [23], we found that current and former smokers had a significantly higher incidence of obesity compared to nonsmokers. However, findings from other published studies that examined the relationships between smoking and BMI or prevalence of obesity have been inconsistent [12,24]. Clarification of the mechanism that explains this association is of considerable interest.

### Study strengths and limitations

The study's sample size, one of its strengths, was large enough to estimate incidence for subgroups and to ensure sufficient statistical power to detect differences across groups. Second, as the largest population-based telephone survey of adults in the United States, BRFSS allows us to obtain incidence estimates that represent all 50 states, the District of Columbia, and three US territories.

The findings in this report are subject to several limitations. First, our estimates are based on a cross-sectional survey rather than following people over time, and this limited our ability to distinguish people who were truly incident cases from those who had been obese in the past, but subsequently lost weight and then regained weight during the previous year (recurrent cases of obesity). We also assumed that the risk factors assessed in 2009 accurately reflected risk-factor status in 2009, and that these risk factors did not differ between incident and recurrent cases of obesity. Second, BMIs were based on reported weight and height, and it is widely known that these estimates, particularly among people with high BMIs, are underestimates [25,26]. Previous studies have found, however, that recalled past weight is strongly correlated with measured weight and that self-reported weight change is reliable [27-29]. Although it is likely that biases in self-reported current and previous weights are correlated, this has not been documented, and our findings need to be confirmed by studies that include measured weights and heights. Third, the survey lacks complete dietary intake data, so we were not able to include all dietary behavioral factors or calorie intake in our modeling analyses. Fourth, the BRFSS excludes people who do not have landline telephones. Because adults who live in wireless-only households tend to be younger, male, Hispanic, binge drinkers, or current smokers, and have lower incomes [30], our incidence estimates may not be generalizable to the entire US population. Based on Council of American Survey and Research Organizations (CASRO) guidelines, the median response rate (percentage of all eligible people who completed interviews) in 2009 was only 52.5% (range: 37.9%- 66.9%), possibly resulting in biased estimates [31].

## Conclusions

In conclusion, we found that the 2009 incidence of obesity was 4% per year and the incidence of extreme obesity was 0.7% per year. There were variations in the incidences of obesity and extreme obesity across socio-demographic groups and populations with certain behavioral factors. The high incidence of obesity underscores the importance of implementing effective policy and environmental intervention strategies in the general population. Given the significant differences in incidence across subgroups, it is possible that additional emphasis should be given to younger adults, women, minorities, and adults with lower education. Our study supports the use of physical activity as a prudent initial step to obesity prevention, but both environmental and policy approaches are needed to prevent weight gain.

## Competing interests

The authors declare that they have no competing interests.

## Authors' contributions

LP participated in the design of the study, performed the statistical data analyses, drafted and revised the manuscript. BS and DSF participated in the design of the study and revision of the manuscript. CG and SP participated in the revision of the manuscript. All authors read and approved the final manuscript.

## Disclaimer

The findings and conclusions in this report are those of the authors and do not necessarily represent the official position of the Centers for Disease Control and Prevention.
